# Impact of Sainfoin (*Onobrychis viciifolia*) Pellets on Parasitological Status, Antibody Responses, and Antioxidant Parameters in Lambs Infected with *Haemonchus contortus*

**DOI:** 10.3390/pathogens11030301

**Published:** 2022-02-27

**Authors:** Michaela Komáromyová, Daniel Petrič, Katarína Kucková, Dominika Batťányi, Michal Babják, Michaela Urda Dolinská, Alžbeta Königová, Daniel Barčák, Emília Dvorožňáková, Klaudia Čobanová, Zora Váradyová, Marián Várady

**Affiliations:** 1 Institute of Parasitology, Slovak Academy of Sciences, Hlinkova 3, 040 01 Košice, Slovakia; komaromyova@saske.sk (M.K.); babjak@saske.sk (M.B.); dolinska@saske.sk (M.U.D.); konig@saske.sk (A.K.); barcak@saske.sk (D.B.); dvoroz@saske.sk (E.D.); 2 University of Veterinary Medicine and Pharmacy in Košice, Komenského 73, 041 81 Košice, Slovakia; petric@saske.sk (D.P.); kuckova@saske.sk (K.K.); 3 Centre of Biosciences of Slovak Academy of Sciences, Institute of Animal Physiology, Šoltésovej 4-6, 040 01 Košice, Slovakia; mravcakova@saske.sk (D.B.); boldik@saske.sk (K.Č.)

**Keywords:** antibody response, antioxidant status, abomasal adult worms, egg hatch test, fecal egg counts, scanning electron microscopy

## Abstract

Our study analyzed the parasitological status, antibody responses, and antioxidant parameters of lambs experimentally infected with a gastrointestinal nematode during the consumption of sainfoin pellets (SFPs) for 14 d. Twenty-four lambs infected with *Haemonchus contortus* were separated into two groups: untreated animals (control) and animals treated with SFPs (600 g dry matter/d). SFP treatment began on day (D) 30 *post*-infection. The number of eggs per gram (EPG) of feces was quantified on D18, D23, D26, D30, D33, D37, D40, and D44. The mean reductions in EPG on D40 and D44 were 33.6 and 36.7%, respectively. The number of abomasal worms was lower for the SFP than the control group (*p* < 0.05). SFP treatment did not significantly affect either the total or the local antibody response (*p* > 0.05). The blood activity of glutathione peroxidase was affected by the treatment (*p* < 0.022). Adult worms were selected for scanning electron microscopy after necropsy, but surface structures of adult *H. contortus* females did not differ between the groups. The treatment of lambs with SFPs directly affected the dynamics of infection, probably indirectly by mobilizing the antioxidant defensive system and antibody response thus improving animal resistance.

## 1. Introduction

The demand for animal products is growing worldwide with the increase of the human population. The intensification of livestock production leads to environmental burdens such as increased use of chemicals and drugs to eliminate the transmission of etiological agents and the pressure on systems of livestock production to ensure feed of sufficient quality [1]. Chemoprophylaxis is currently the most effective method of preventing severe losses in livestock caused by gastrointestinal nematodes (GINs). However, without consideration of maintaining a certain refugium, these practices can be considered inappropriate and unsustainable in the long term, because resistance to any new drug may develop within 10 years [2]. Among the GINs of small ruminants, the highly pathogenic *Haemonchus contortus* represents a major threat in animal husbandry. *H. contortus* is a common cause of production losses and even deaths in livestock due to its blood-sucking behavior and rapid reproduction [3]. Parasitism increases the demands on the resources of proteins and calories from the repair of damaged tissues and the production of immune cells and mediators of the immune response [4,5]. The nutritional status of the host has been considered an important factor influencing the pathogenesis of parasitic diseases and the relationship between the host and parasite [6]. Controlling the intensity of parasitic infection can improve nutritional deficits and the overall health of the animal [7]. 

The antiparasitic activity of polyphenols not only directly affects parasites [7] but also indirectly modulates the immune response [8]. Oxidative stress is known to occur when reactive oxygen species or free radicals exceed the detoxification capacity of antioxidants [9]. GIN infections may induce the production of reactive oxygen species, which may damage the parasites but generate oxidative stress by the formation of reactive oxygen molecules (e.g. superoxide radicals, hydroxyl radicals, and hydrogen peroxide) [10]. Feed supplementation with medicinal herbs containing bioactive compounds may attenuate the adverse effects of parasite infection by stimulating endogenous antioxidant defense systems and helping trigger local immune responses in the abomasal mucosa of small ruminants [11,12]. Polyphenols are the most common antioxidants known for their immunomodulatory and anti-inflammatory activities [13]. One possible way to control and reduce infection caused by GINs is the use of tanniferous forages containing condensed tannins (CTs) with direct anthelmintic effects on GINs, which can lead to a substantial decrease in the excretion of nematode eggs and reduce the contamination of pastures [14]. Electron microscopy has found that the direct anthelmintic effects of CTs can damage the cuticle and digestive tissues of larval or adult parasites [15]. The legume sainfoin, *Onobrychis viciifolia*, has a high content of CTs, especially proanthocyanidins, and its consumption disturbs different life stages of parasite life cycles [16,17]. The benefits of sainfoin use also include a reduced requirement for chemical fertilizers because of its biological fixation of nitrogen, high palatability for ruminants, feeding value, reduction of ruminal methane emission, anti-bloating effects, and nitrogen excretion in urine and feces [8]. 

The process of pelleting sainfoin (i.e. high temperature and pressure) does not affect the polyphenolic bioactivities linked to antioxidative properties [18], so we hypothesized that sainfoin pellets (SFPs) may also contribute to changing the course of infection caused by *H. contortus* in lambs by lowering fecal egg counts (FEC) and the number of adult worms in the abomasum and by improving the immune response and antioxidant status. Our goals were therefore to investigate the effect of SFPs on the parasitological status, immune response, and antioxidant parameters of lambs experimentally infected with *H. contortus.*

## 2. Results

### 2.1. Fecal Egg Count (FEC) and Weight Gains

**Figure 1:**
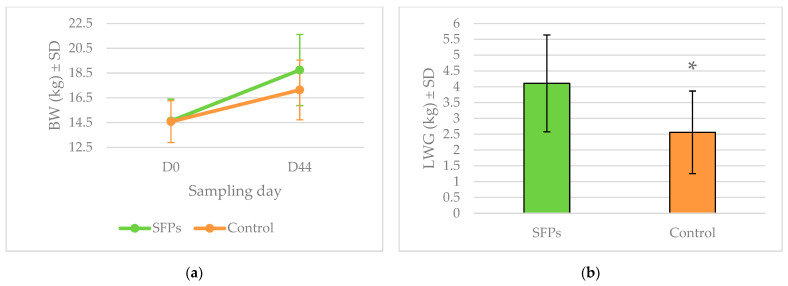
(**a**) Mean body weight (BW) and (**b**) mean live-weight gain (LWG) (*, *p* < 0.05).

Lamb total body weight (BW) did not differ significantly between groups, but live-weight gain (LWG) was significantly lower (*p* < 0.05) in the control than in the SFP treated group. Changes in BW during the experimental period and differences in LWG at the end of the experiment are shown in Figure 1.

**Figure 2:**
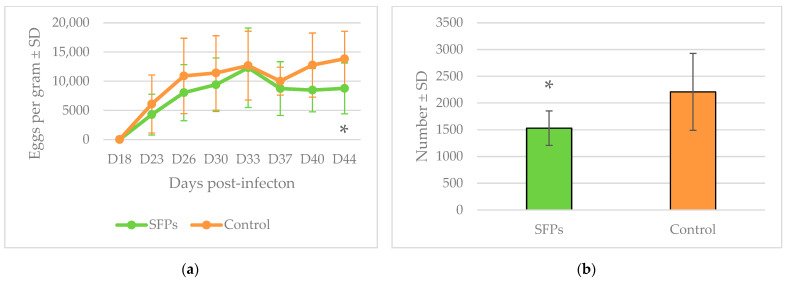
(**a**) Mean fecal egg counts for the groups of lambs infected with *Haemonchus contortus* and treated or not with SFPs (*, *p* < 0.05). (**b**) Mean number of *H. contortus* worms in the abomasum at the end of the experiment (*, *p* < 0.05).

Reduction in egg shedding (13.7%) in the SFP group was first noticeable on day 37 (D37). A fecal egg count reduction test (FECRT) confirmed reductions of 33.6 and 36.7% (*p* < 0.05) on D40 and D44, respectively, in the SFP versus the control group. (Figure 2a). The number of abomasal worms after the necropsy was 30.6% lower in the SFP versus the control group (*p* < 0.05, Figure 2b). Female fecundity (per worm) did not differ significantly between the groups. 

### 2.2. Egg Hatch Test (EHT)

The aqueous extracts of the SFPs had a significant ovicidal effect on *H. contortus*, with mean ED_50_ and ED_99_ of 4.67 and 25.33 mg/mL, respectively (Table 1). Each concentration of the SFP extract tested affected the proportion of hatched eggs (*p* < 0.001).

### 2.3. Antibody Responses 

The treatment did not significantly affect either the total (Table 2) or local (Table 3) antibody response of the treated animals (*p* > 0.05). Mean serum IgA concentrations and eosinophil peroxidase (EPx) increased over time (*p* < 0.035 and *p* < 0.001, respectively), both peaking in both groups on D44 (Table 2).

### 2.4. Antioxidant Status 

Time and the interaction between treatment and time affected total antioxidant capacity (TAC, *p* < 0.004) (Table 4), and treatment and time affected glutathione peroxidase (GPx) activity (*p* < 0.022 and *p* < 0.001, respectively). GPx activity from D30 to D44 was higher for the control than the SFP group. The serum malondialdehyde (MDA) concentration was significantly influenced by time in both groups (*p* < 0.001). 

### 2.5. Scanning Electron Microscopy (SEM)

**Figure 3:**
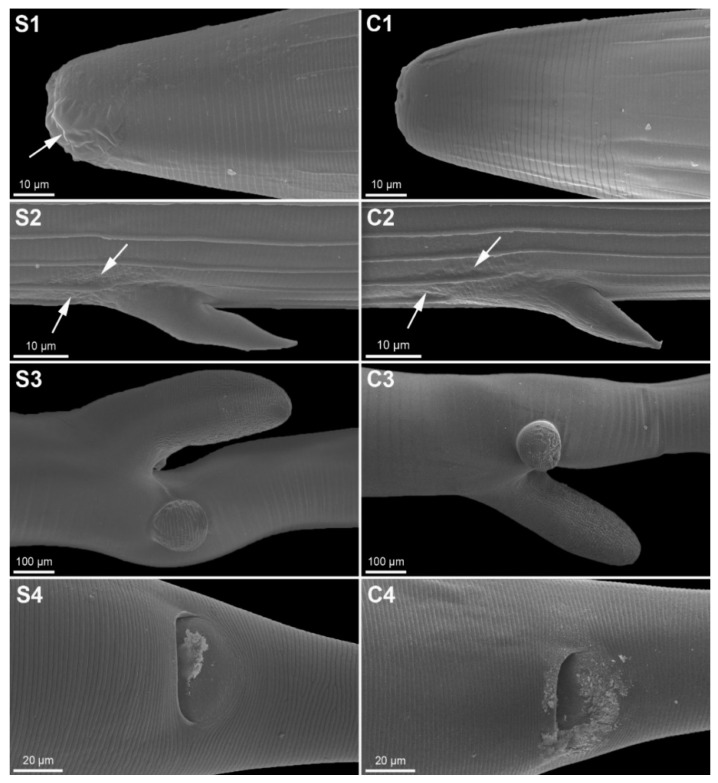
Scanning electron micrographs of adult female *Haemonchus contortus* isolated from the abomasa of lambs fed with SFPs (**S1**–**S4**) and from the control animals (**C1**–**C4**). Micrographs show the cephalic region with a detached (arrow) cuticle in the apical part (**S1**) compared with a control individual (**C1**), a cervical papilla with slight protuberances around its base (**S2**,**C2**), the smooth surface of the posterior part of the body with the vulvar flap and lateral button (**S3**,**C3**), and the terminal part of the body with remnants of debris near the anal pore (**S4**,**C4**).

The surface structures of adult female *H. contortus* examined by SEM (Figure 3) did not differ significantly between the animals fed with SFPs (*n* = 12) and the control group (*n* = 6). The only obvious difference in the treated group was the detachment of the cuticle in the cephalic region (Figure 3(S1)), but only one of the 12 specimens exhibited such damage. The cuticle in the middle part of the body in both groups possessed only physiological longitudinal ridges (Figure 3(S2,C2)), with no wrinkled cuticular surface on any of the worms (Figure 3). Slight protuberances (arrow) were common around the bases of the cervical papillae, but both the treated (Figure 3(S3)) and control (Figure 3(C3)) specimens had this trait. Some debris was near the anal pore of a few specimens in the SPF and control groups (Figure 3(S4,C4)).

## 3. Discussion

We predicted that lambs infected with *H. contortus* and treated with SFPs would have noticeable reductions in FEC and total abomasal worm counts compared to the control lambs. We thus also expected better overall health conditions, demonstrated as a higher LWG in the treated lambs. Tanniferous forages can increase the LWGs of sheep infected with *H. contortus* [19], consistent with our results at the end of the experiment. Our results indicated that the SFP group had a lower parasitic load based on differences in LWG between the groups. This lower load was confirmed not only by significant differences in LWG between groups but also by FECRs and mean worm counts at necropsy. Lower growth rates are generally considered to be an indicator of parasitism in lambs infected with GINs, but plant polyphenols, especially flavonoids, can improve the growth of animals and the quality of animal products [20]. We previously reported that dried plant nutraceuticals containing different amounts of flavonoids and phenolics may [21] or may not [22,23,24] influence the growth parameters of infected lambs. The SFPs in the present experiment contained 4.62 mg QE/g DM flavonoids. We previously reported flavonoid range of 10–40 g/kg DM in herbal nutraceuticals [22,25,26]. 

The effects on BW and LWG probably depend on the source and variety of polyphenols and on the combination of the multitarget complex bioactive compounds that can work synergistically and antagonistically [26]. 

The FEC and worm burden during the 14-day administration of SFPs were reduced by 36.7 and 30.6%, respectively, as direct nematocidal effects. Tanniferous feeds can directly affect worm biology and modulate the epidemiology of GIN infections as pharmacological-like processes [16,27]. The direct anthelmintic effect of the SFPs on the reduction of FEC and the number of adult worms in our experiment is consistent with several other trials [28,29,30]. Sainfoin diets, (i.e. dried and ensiled) consumed in these trials for 16 d by lambs infected with *H. contortus* reduced the number of adult worms by 47 and 49%, respectively, and the FEC was reduced by 58 and 48%, respectively [28]. The inclusion of sainfoin (20%) in ground lucerne pellets to the diets of lambs infected with *H. contortus* for 30 d reduced both FEC (54%) and the number of adult worms (13%) [29]. A similar study with calves receiving sainfoin pellets for 42 d *post*-infection reported reduced parasite populations of *Ostertagia ostertagi* by 50% [30]. In our experiment, the reductions in FEC and the number of adult parasites were lower compared to the above studies. We administered the SFP diet to a group of animals from 30 d after the experimental infection when the parasites had developed into the adult stage, whereas the sainfoin diet in the other studies [28,29,30] was administered to the animals from, or several days before, the experimental infection. The prolonged administration of the sainfoin diet could thus also have affected the developing larval stages of the parasites, which could increase the efficiency of the diet.

The aqueous extract of the SFPs in our study had a strong ovicidal effect (>70%) at concentrations *≥* 6.25 mg/mL in the EHT. Dried tanniferous plants used as fodder or in in vitro tests can directly affect nematode species and their stages [31,32,33], probably due to the size and the percentage of flavan-3-ols of prodelphinidin-type CTs that influence anti-parasitic activity against nematodes [34]. The anthelmintic activity of CTs against GINs may be increased by the addition of flavonoids [35], and sainfoin itself contains beneficial flavonoids that interfere with the biology of *H. contortus* [36], consistent with our results. The use of dry medicinal plants with high contents of flavonoids against *H. contortus* in vivo can have an indirect anthelmintic effect that can contribute to an increase in the resistance of lambs to nematode infection [22,23,24]. The anthelmintic effects of the plant extract in vitro and the sensitivity of the methodological procedures used should be taken into account for the overall evaluation of plants with bioactive components [37,38].

The level of IgA antibodies against *H. contortus* in our experiment increased significantly over time after infection in both groups. A study with a longer (six weeks) consumption of sainfoin hay described an enhanced local immune response to *Trichostrongylus colubriformis* and a significant reduction in the FEC in sheep [39]. IgA activity against the parasite *Teladorsagia circumcincta* in sheep has also been associated with a reduction in fertility and the length of adult parasites [40]. Low IgA levels in infected animals are associated with low-intensity infections or with heavy infections, when most of the mucosal IgA is bound to parasite antigens [41]. The higher levels of IgA and higher EPx activity in our experiment responded strongly to infection and were correlated with decreased FECs of the animals. The EPx enzyme is involved in killing parasites and implicates eosinophils in the direct control of parasitic infections [42,43]. Our results suggest that a longer time (>14 d) of consumption of sainfoin pellets by infected animals is needed to produce a prominent antibody response to the treatment. Tanniferous plants have immunomodulatory effects against GINs, but helminths secrete an extremely broad spectrum of immunoregulatory molecules to ensure survival inside their host, which can also have consequences on the immune responses of host animals [44]. 

Our results indicated that the activity of GPx in the blood decreased continually from the beginning of the SFP treatment (i.e., 30–37 d *post*-infection). The serum level of MDA, as a marker of oxidative stress, was not affected by the treatment, but lipid oxidation in the SFP treatment tended to decrease. SFPs decreased blood GPx activity from D23 indicating that lower antioxidant activity is needed for lambs treated with SFPs to maintain the same oxidative status. These findings are consistent with our prior results using medicinal plants with a wide range of bioactive compounds [22,23,24] that possess the antioxidant potential and are supplements for diseases associated with oxidative stress [45]. The antioxidant status of lambs probably closely depends on the efficiency of absorption, concentration, and metabolic transformation of phytochemicals and the environment [46]. Some medicinal plants are often characterized as poisonous and medicinal, but each of the medicinal plants, both as mixtures and SFPs, have been tested by in vitro rumen fermentation without adverse effects on the rumen microbiome [21,22,23,24]. Another benefit of medical treatment is the use of plants as substitutes for synthetic drugs to which parasites have developed resistance in the past. 

SEM recently became a tool to demonstrate the possible anthelmintic effects of nutraceutical plants in multiple studies [47,48,49]. Studies on ultrastructural changes have mostly been conducted under in vitro conditions with various plants. The effects of *Biophytum petersianum* on *H. contortus*, such as wrinkling on the cuticular surface followed by exfoliation of the tegument on the tail section, have been described [50]. The main changes to female *H. contortus* after in vitro exposure to SFPs were on the cuticle and in the vulval region and buccal area [27]. Less data, however, are available on the in vivo effects of herbal substances on GINs. The damaging effects of sainfoin on third-stage larvae [51] and adult *H. contortus* [52] have been described using transmission electron microscopy, such as the detachment of cuticles and changes in intestinal and muscular cells that can negatively affect the viability of parasites. Ultrastructural changes after in vivo treatment with CTs have mostly been reported as local cuticular damage and changes on buccal capsules [30,53]. One of the most common features in our worms were protuberances around the bases of the cervical papillae, which were not mentioned in other studies, but the control group also had this feature. The cuticle across parasites from both groups were smooth with physiological longitudinal ridges. Only one more pronounced cuticular damage (Figure 3(S1)) was observed in worms from the SFP group, but the sample size was too small for confirming the overall effect. Cuticular wrinkling has been reported in the majority of studies of ultrastructural changes [48,49,54,55]. Aggregates near the buccal capsule, vulva, and anus have been described as a change in treated worms [53], but we also found these traits in the control group. A correlation between the length of exposure to CTs and ultrastructural changes that were more prominent after 77 d of treatment in comparison with 28 d has also been reported [30]. We cannot confirm the effect of SFPs on the surface structures of *H. contortus* after 14 d of treatment, because we did not find any of the changes reported in previous SEM studies.

## 4. Materials and Methods

### 4.1. Ethics Statement

This study was conducted following the guidelines of the Declaration of Helsinki and national legislation in the Slovak Republic (G.R. 377/2012; Law 39/2007) for the care and use of research animals. The experimental protocol was approved by the Ethical Committee of the Institute of Parasitology of the Slovak Academy of Sciences on 22 November 2020 (protocol code 2020/21).

### 4.2. Experimental Design, Diets, and Experimental Infection

Twenty-four female Improved Valachian lambs, all 3–4 months old with no grazing history and a mean weight of 14.58 ± 1.689 kg, were housed in common stalls on a sheep farm (PD Ružín–Ružín farm, Kysak, Slovakia) with free access to water. The animals were dewormed with the recommended dose of albendazole (Albendavet 1.9% susp. a.u.v, DIVASA-FARMAVIC S.A., Barcelona, Spain) 14 d before the start of the trial and were kept indoors to maintain parasite-free conditions. The diet of the animals included meadow hay ad libitum and 300 g of dry matter (DM)/d of a commercial concentrate, Mikrop ČOJ (MIKROP, Čebín, Czech Republic). After the acclimation period, the lambs were infected with 5000 third-stage larvae of the *H. contortus* MHCo1 strain susceptible to anthelmintics [22]. The lambs were divided into two groups of twelve animals each (one stall per group) on D30 after infection when all parasites had matured to the adult stage: animals treated with sainfoin pellets (SFPs, 600 g DM/d/animal) and untreated animals (control, meadow hay, 600 g DM/d/animal). SFPs were obtained from a commercial source (NATURE´S BEST, EQUOVIS GmbH, Münster, Germany). This treatment scheme continued for 14 d. Animals were housed in collective stalls according to dietary treatment (*n* = 12/stall) and adequate access to water and feeder space was provided for each animal. Feeders were placed and designed to allow easy access to the complete ratio for all lambs in each group and also to avoid wasting feed. One-sided access to the feeding troughs allowed 40–45 cm per lamb. The animals completely consumed the concentrate and SFPs and no food rejection was noted. All animals were humanely slaughtered at the end of the trial (D44) following the rules of the European Commission (Council Regulation 1099/2009) for slaughtering procedures [56]. 

### 4.3. Chemical Tests of Sainfoin Pellets (SFPs) 

Chemical tests for the screening of SFP bioactive compounds were carried out in aqueous extracts (saponins, terpenoids) or ethanolic extracts (tannins, alkaloids, flavonoids, steroids) (Table 5) using standard procedures [57].

The total phenolic and flavonoid content in the SFP extract was 13.86 mg TAE/g DM and 4.62 mg QE/g DM, respectively. The SFP extract was prepared as described earlier by Petrič et al. (2020). The sainfoin pellets (10 g) were crushed and mixed with 100 ml of ethanol (70%) using a magnetic stirrer at room temperature for 24 h. Thereafter, the extract was filtered throughout Whatman filter paper No. 1 to obtain extract without plant particles. The residue was re-extracted twice and the pooled extract was used for the analysis of total phenolics and flavonoids content. The concentration of phenolic compounds in the extract was determined by Folin-Ciocalteu colorimetric assay [58] and expressed as mg tannic acids equivalents per gram of dried weight sample (mg TAE/g DM). The total flavonoids content in the SFP extract was determined [59] and results were expressed as mg quercetin equivalents per gram of dried weight sample (mg QE/g DW).

### 4.4. Fecal Egg Count and Animal Weighing

Fecal samples were collected rectally on D18, D23, D26, D30, D33, D37, D40, and D44 *post*-infection. The number of eggs per gram (EPG) of feces was quantified using the McMaster method previously described [60]. The lambs were weighed on D0 and D44. 

### 4.5. Counts of Adult Worms

After slaughtering on D44, a helminthological necropsy was performed to count the total number of worms. The abomasum of each animal was removed and dissected, and the abomasal contents were washed with warm physiological saline and emptied into a jar. The contents of the jar were mixed continuously to prevent the clustering of nematodes, and washings were brought up to a volume of two liters with water. Two 40-mL aliquots were collected and fixed with helminthological iodine, and the recovered *H. contortus* adults were counted and classified as male or female. Female fecundity (egg production per worm) on D44 was then calculated as EPG on D44 in both groups divided by the number of female worms found at necropsy in the corresponding groups.

### 4.6. Egg Hatch Test 

An EHT was performed to determine the ovicidal activity of the aqueous extract prepared from ground SFPs, as described previously [25]. Eggs of susceptible *H. contortus* were isolated from feces freshly collected from the rectums of lambs from the untreated control group. Final concentrations of the SFP aqueous extracts used in the EHT were 50, 25, 12.5, 6.25, 3.125, and 1.563 mg/mL. The EHTs were performed in six independent determinations. The concentrations of extract that prevented 50 and 99% of the eggs from hatching (median effective dose, ED_50_) were established, and ovicidal activity was calculated. 

### 4.7. Antibody Responses 

Mucus samples obtained from the abomasa were used to determine the levels of total IgA. Mucus was collected after necropsy by light scraping of the abomasal mucosa using a glass slide. The mucus was diluted in a buffer (pH 7.1; Na2HPO4 0.1 M; NaCl 0.05 M; NaN3 3 mM; PMSF 1 mM; EDTA 5 mM) at the rate of 2.5 mL/g mucus, homogenized, and centrifuged at 18,000× g at 4 °C for 30 min [61]. The supernatant was collected and stored at −20 °C until analysis. Adult worms were harvested from the abomasa of the infected lambs. The excretory/secretory products (ESPs) of adult *H. contortus* were obtained as described by [23], with some modifications. Freshly collected adults were washed five times in warm phosphate-buffered saline (pH 7.4). The parasites were then transferred at a concentration of 500 adults/20 mL to RPMI 1640 medium containing 100 U/mL penicillin and 100 µg/mL streptomycin (Sigma-Aldrich, Hamburg, Germany) and incubated in 5% CO_2_ at 37 °C for 4 h. The worms were then removed, and the medium containing the ESPs was centrifuged at 20,000× *g* at 4 °C for 15 min. The supernatant with the ESPs was centrifuged in 3000 MWCO VIVASPIN tubes (Sartorius, Goettingen, Germany) at 4000× *g* at 4 °C for 60 min and stored at −20 °C. The ESP protein concentration was measured using the Bradford protein assay (Bio-Rad Laboratories, Munich, Germany). The levels of total IgA in the mucus and serum and specific IgG and IgM against *H. contortus* in the serum were determined using a rabbit anti-sheep IgA antibody (Bethyl Laboratories, Inc., Montgomery, AL, USA) or an ESP antigen in an indirect enzyme-linked immunosorbent assay (ELISA), similar to a previously described method [62]. *H. contortus* ESPs were diluted to a final concentration of 5 µg/mL for IgG and IgM. Anti-sheep IgA antibody for serum and mucus IgA was diluted to concentration 1:500. The serum samples were diluted 1:100, the mucus samples were diluted 1:10, and horseradish peroxidase-conjugates rabbit anti-sheep IgG (Sigma-Aldrich, Hamburg, Germany), rabbit anti-sheep IgM H&L (Abcam, Cambridge, MA, USA), and rabbit anti-sheep IgA (Bio-Rad Laboratories, Inc., Kidlington, UK) were diluted 1:2000, 1:50,000, and 1:10,000, respectively. The results are expressed as optical densities measured using an Apollo 11 LB913 Elisa absorbance reader (Berthold Technologies GmbH & Co. KG, Bad Wildbad, Germany). Sheep eosinophil peroxidase (EPX) level was measured using an ELISA kit (MyBioSource Ltd., San Diego, CA, USA) with a sensitivity of 1.0 ng/mL. 

### 4.8. Antioxidant Parameters

The activity of glutathione peroxidase (GPx) in the blood, total antioxidant capacity (TAC) in the serum, and the serum concentration of malondialdehyde (MDA) were determined as previously described [11].

### 4.9. Scanning Electron Microscopy (SEM)

Worms were randomly collected during necropsy from the SFP treated group and control animals. Adult specimens selected for SEM were rinsed in saline, fixed in a 4% formaldehyde solution near the boiling point, and stored in 70% non-denatured ethanol. Each specimen was cut into three parts (anterior, middle, and posterior), which were dehydrated in an ascending series of concentrations of ethanol (80, 90, 96, and 100%; 15 min. in each concentration), chemically dried in 1,1,1,3,3,3-hexamethyldisilazane (Merck, Darmstadt, German), and gold-sputtered using a JEOL JFC 1300 auto fine coater (JEOL Ltd., Tokyo, Japan). The SEM micrographs were captured using a JEOL JSM 6510LA.

### 4.10. Statistical Analysis

We evaluated the efficacy of the SFP treatment using a FECRT (%) and the formula recommended by the World Association for the Advancement of Veterinary Parasitology (WAAVP) [60]: (1 − [T ÷ C]) × 100, where T is the arithmetic mean EPG for the SFP group 14 d after treatment and C is the arithmetic mean EPG for the control group at D44. A logit model of regression analysis was applied on egg hatch data to express an ED_50_ and ED_99_ concentration for each EHT. The differences between egg production (EPGs) in the groups on different sampling days after treatment, differences between the number of worms in the abomasum in the groups of animals, the ovicidal effect on parasite eggs in the EHT, and differences between the levels of total IgA in the mucus were assessed using Student’s *t*-tests. The antibody responses and antioxidant parameters were statistically analyzed using analyses of variance (ANOVAs) (GraphPad Prism 8; GraphPad Software, Inc., San Diego, CA, USA) as a repeated-measures mixed model representing the two groups and sampling days. Differences between the groups were identified using a two-way ANOVA. Results were considered significant at *p* < 0.05.

## 5. Conclusions

The treatment of infected lambs with SFPs for 14 d improved the parameters of production and directly affected the dynamics of infection without strong antibody responses of the treated animals. The mobilization of the antioxidant defensive system, however, suggested that the animals were able to protect themselves against the new oxidative conditions. Dietary antioxidants in the form of SFPs were available to sustain the antibody response and thus indirectly improved the resistance of the animals against *H. contortus.*

## Figures and Tables

**Table 1:** Egg hatching and ovicidal activity of tested concentrations of SFPs extract in an EHT.

Concentration (mg/mL)	Hatching (%)	Ovicidal Effect (%)	*p*
50	5 ± 7.42	95	<0.001
25	4 ± 6.33	96	<0.001
12.5	11 ± 14.47	89	<0.001
6.25	26 ± 28.74	74	<0.001
3.125	76 ± 12.25	24	<0.001
1.563	88 ± 3.71	12	<0.001
0	98 ± 1.095	-	-

Mean egg hatching ± SD (n = 6).

**Table 2:** Total antibody response in the blood serum of infected lambs treated or not with SFPs.

	Day	SFPs	Control	SD	*p*		
					Treatment (T)	Time	T × Time
IgG (OD)	23	0.357	0.361	0.0773	0.611	0.837	0.987
	30	0.397	0.374	0.1512			
	37	0.402	0.385	0.1207			
	44	0.390	0.369	0.0763			
IgA (OD)	23	0.405	0.401	0.0771	0.475	0.035	0.704
	30	0.398	0.411	0.0651			
	37	0.417	0.376	0.0751			
	44	0.467	0.451	0.0580			
IgM (OD)	23	0.458	0.509	0.1048	0.453	0.175	0.821
	30	0.479	0.479	0.1265			
	37	0.489	0.535	0.1243			
	44	0.566	0.555	0.1169			
EPx (ng/mL)	23	22.7	18.2	13.70			
	30	28.6	25.3	15.76			
	37	44.3	37.0	20.29	0.509	0.001	0.814
	44	47.6	51.5	16.02			

IgG, immunoglobulin G; IgA, immunoglobulin A; IgM, immunoglobulin M; OD, optical density; EPx, eosinophil peroxidase; SD, standard deviation.

**Table 3:** Local antibody response in the abomasal mucus of lambs treated or not with SFPs.

	Day	SFP	Control	*p*
IgA (OD)	44	0.401 ± 0.0928	0.441 ± 0.0916	0.280

OD, optical density.

**Table 4:** Antioxidant status in blood serum of infected lambs treated or not with SFPs.

	Day	SFPs	Control	SD	*p*		
					Treatment (T)	Time	T × Time
TAC (mmol/L)	0	0.578	0.527	0.062	0.919	0.004	0.004
	23	0.536	0.547	0.060			
	30	0.522	0.552	0.110			
	37	0.471	0.518	0.054			
	44	0.509	0.483	0.065			
GPx (U/g Hb)	0	486.4	506.7	115.3	0.022	0.001	0.857
	23	518.2	530.7	117.8			
	30	380.4	434.0	97.16			
	37	254.5	316.9	75.62			
	44	175.3	224.5	72.15			
MDA (µmol/L)	0	0.265	0.269	0.037	0.060	0.001	0.599
	23	0.235	0.247	0.033			
	30	0.242	0.269	0.028			
	37	0.241	0.248	0.041			
	44	0.273	0.297	0.035			

TAC, total antioxidant capacity; GPx, blood glutathione peroxidase; Hb, hemoglobin; MDA, malondialdehyde; T, treatment; SD, standard deviation.

**Table 5:** Chemical composition of the aqueous and ethanolic extracts of the sainfoin pellets (SFPs).

	Tannins	Saponins	Alkaloids	Terpenoids	Flavonoids	Steroids
SFPs	+	−	−	+	+	−

+, bioactive compounds present; −, bioactive compounds not present.

## Data Availability

Data are available upon reasonable request to the corresponding author.
